# Radiomics predicts the prognosis of patients with locally advanced breast cancer by reflecting the heterogeneity of tumor cells and the tumor microenvironment

**DOI:** 10.1186/s13058-022-01516-0

**Published:** 2022-03-15

**Authors:** Xuanyi Wang, Tiansong Xie, Jurui Luo, Zhengrong Zhou, Xiaoli Yu, Xiaomao Guo

**Affiliations:** 1 Department of Radiation Oncology, Fudan University Shanghai Cancer Center, 270 DongAn Road, Shanghai, 200032 China; 2 Department of Radiology, Fudan University Shanghai Cancer Center, Shanghai, 200032 China

**Keywords:** Radiomics, Locally advanced breast cancer, Tumor heterogeneity, Tumor microenvironment

## Abstract

**Background:**

This study investigated the efficacy of radiomics to predict survival outcome for locally advanced breast cancer (LABC) patients and the association of radiomics with tumor heterogeneity and microenvironment.

**Methods:**

Patients with LABC from 2010 to 2015 were retrospectively reviewed. Radiomics features were extracted from enhanced MRI. We constructed the radiomics score using lasso and assessed its prognostic value. An external validation cohort from The Cancer Imaging Archive was used to assess phenotype reproducibility. Sequencing data from TCGA and our center were applied to reveal genomic landscape of different radiomics score groups. Tumor infiltrating lymphocytes map and bioinformatics methods were applied to evaluate the heterogeneity of tumor microenvironment. Computational histopathology was also applied.

**Results:**

A total of 278 patients were divided into training cohort and validation cohort. Radiomics score was constructed and significantly associated with disease-free survival (DFS) of the patients in training cohort, validation cohort and external validation cohort (*p* < 0.001, *p* = 0.014 and *p* = 0.041, respectively). The radiomics-based nomogram showed better predictive performance of DFS compared with TNM model. Distinct gene expression patterns were identified. Immunophenotype and immune cell composition was different in each radiomics score group. The link between radiomics and computational histopathology was revealed.

**Conclusions:**

The radiomics score could effectively predict prognosis of LABC after neoadjuvant chemotherapy and radiotherapy. Radiomics revealed heterogeneity of tumor cell and tumor microenvironment and holds great potential to facilitate individualized DFS estimation and guide personalized care.

**Supplementary Information:**

The online version contains supplementary material available at 10.1186/s13058-022-01516-0.

## Background

Breast cancer is the most commonly diagnosed cancer and the leading cause of death among females worldwide [1]. Locally advanced breast cancer (LABC) consists of breast cancer with different stages and prognoses and requires comprehensive treatment [2]. Neoadjuvant chemotherapy (NAC) is the standard treatment for LABC and radiotherapy (RT) is an important component of comprehensive treatment for LABC. Prospective randomized trials, meta-analyses, and observational data have demonstrated that postoperative radiotherapy (PORT) reduces locoregional recurrences (LRR), distant recurrences, and breast cancer mortality (BCM) in pN + patients [3, 4]. Given the treatment complexity and tumor heterogeneity, it is difficult to precisely predict the prognosis of LABC. At present, prognostic assessment for these patients is mainly dependent on the clinical and pathological evaluations in terms of TNM staging and molecular subtypes. Therefore, the development of a new method to assess the prognosis of patients with LABC undergoing neoadjuvant chemotherapy and postoperative RT is important.

Radiomics is a noninvasive method that extracts quantitative features from medical imaging in a high-throughput manner [5], and much progress has been made in this new and promising field. Radiomics have been used to guide cancer management, including diagnosis, prognosis prediction, tumor staging, and treatment response evaluation [6–9]. Recent studies have demonstrated that radiomics can improve or outperform the existing method in terms of tumor diagnosis and prognosis prediction. Several publications have suggested that radiomics can predict prognosis in malignant tumors such as pancreatic cancer, gastric carcinoma, breast cancer, and nasopharyngeal carcinoma [10–13]. Indeed, radiomics have been applied to predict the complete pathological remission (pCR) rate for patients with breast cancer receiving NAC [14]. However, data on the use of radiomics in patients with LABC after NAC and PORT (postoperative radiotherapy) are limited, and whether radiomics can be used to predict the survival outcome of patients with breast cancer after NAC and RT is unknown.

Accumulating evidence suggests that imaging subtypes are associated with distinct gene expression patterns [15–18]. Both pro-oncogenic and anti-oncogenic pathways are enriched in different image subtypes, including the mTOR and cell cycle pathway. Previous studies have mainly focused on the association of radiomics and tumor cell heterogeneity. However, the association between radiomics and the tumor microenvironment, which comprises heterogeneous cell types and plays a pivotal role in determining prognosis, remains unclear.

Additionally, computational histopathology has been developed to quantify histopathological patterns in histopathology image slides. Both radiomics and computational histopathology are derived from tumor tissue and extract tumor morphological phenotypes to reveal tumor heterogeneity [19, 20]. Therefore, computational histopathology may be able to reflect morphological changes detected by radiomics and vice versa. However, whether and how computational histopathology could be combined with radiomics remains unknown.

Here, we constructed and validated an MRI-based radiomics score (RS) to assess the DFS of patients with locally advanced breast cancer after NAC and PORT. By incorporating radiomics with genomic and computational histopathological data, we evaluated the association between radiomics and heterogeneity of tumor cells and their microenvironment.

## Methods

### Study population and treatment

**Fig. 1:**
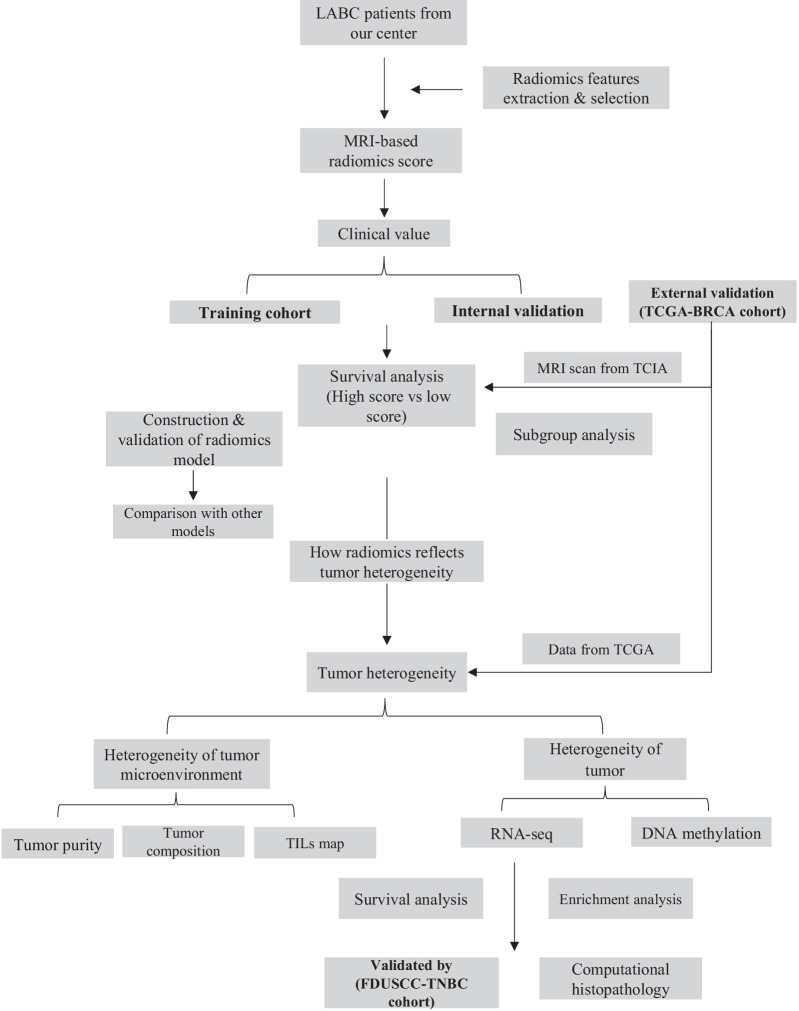
The overview of this study

The design of the study is presented in Fig. 1. The study population comprised a training cohort, validation cohort, and an external validation cohort (TCGA-BRCA cohort).

Training and validation cohorts were collected in Fudan University Shanghai Cancer Center. A total of 441 patients who presented with clinical stage II–III breast cancer and who underwent NAC and PORT from 2010 to 2015 were retrospectively reviewed. The study was approved by the Institutional Review Board, and the reviewed data included clinical, histopathological, and imaging data.

The inclusion criteria of this study are presented in the supplement. Finally, 278 patients were enrolled and were randomly divided at a 1:1 ratio into training and validation cohorts.

The baseline clinical characteristics and pathologic data were derived from patients’ medical records and included age, laterality, cT stage, cN stage, pT stage, pN stage, estrogen receptor (ER) status, progesterone receptor (PR) status, human epidermal growth factor receptor-2 (HER-2) status, treatment response, chemotherapy regimen, and RT plan. Preoperative breast MRI scans were recorded and extracted from the picture archiving and communication system (PACS) in the Department of Radiology in Fudan University Shanghai Cancer Center. The TNM staging for each patient was reclassified according to the eighth edition of the Cancer Staging Manual of the American Joint Committee on Cancer (AJCC)/International Union Against Cancer. The pathological results were reviewed and confirmed by the central laboratory of the Department of Pathology in Fudan University Shanghai Cancer Center. The pCR of the primary tumor after NAC was defined as the eradication of all invasive diseases in the breast and regional lymph nodes.

The systemic treatment, surgery, and RT plan were consistent with the previously published data [21]. The taxane-based PC (paclitaxel and carboplatin) regimen was the most commonly used NAC regimen. Most patients underwent mastectomy and axillary lymph node dissection (ALND). PORT was performed after the completion of adjuvant chemotherapy. Regional nodal irradiation (RNI) was delivered to the ipsilateral supraclavicular region and the infraclavicular region + / − internal mammary lymph nodes (IMNs). The dissected axillary region was excluded from the irradiation fields. The prescription dose for most patients who underwent mastectomy was 50 Gy in 25 fractions. For patients who underwent lumpectomy, a boost of 10 Gy in five fractions was delivered to the lumpectomy cavity. IMNI was given according to the discretion of radiation oncologists, and the prescription dose was 50 Gy in 25 fractions. RT fields were confirmed by reviewing each RT plan.

The external validation cohort (TCGA-BRCA cohort) was collected from The Cancer Imaging Archive (TCIA). Breast cancer cases from TCIA TCGA-BRCA cohort without enhanced MRI or those with had no clear lesions on MRI were excluded. As a result, 91 cases were enrolled.

### Endpoints and follow-up

The primary endpoints of this study were DFS (defined as the interval from the date of curative surgery to disease recurrence, secondary malignancy, death, or the last visit) and RFS (recurrence-free survival, defined as the interval from the date of curative surgery to disease recurrence, death, or the last visit).

Patients treated in our center were followed up every 3 months in the first 2 years, every 6 months in the next 3 years, and once a year after that. Physical examination, laboratory test, and ultrasound of the breast, lymph nodes, and abdominal organs were performed at every follow-up. Radiological imaging such as chest CT and mammogram was performed once a year. Breast MRI was performed when necessary. Follow-up data for the external validation cohorts were obtained from the TCIA.

### MRI acquisition and segmentation

Patients from our center underwent breast MRI in the 4 weeks before NAC. A detailed description of MRI acquisition is presented in the supplement.

MRI imaging segmentation were performed before feature extraction. To obtain the good contrast between tumor and surrounding breast parenchyma, the T1-enhanced MRI digital imaging and communication in medicine (DICOM) images that have been archived in PACS were acquired. The target MRI image was then loaded into personal computer for segmentation. Two experienced radiologists reviewed MRI images respectively. They were blinded to prognosis but aware that these patients were eventually diagnosed as breast cancer. Region of interest (ROI) of tumor was manually segmented along the lesion in every slice by the first reviewer and then reviewed by the second reviewer. The intra- and inter-observer agreement of features extraction was assessed by ICC. We randomly selected 20 patients from our center and calculated ICC of all radiomics features. Inter- and intra-ICC greater than 0.75 represent good agreement between reviewers.

### MRI-based texture analysis

Image preprocessing, tumor segmentation, and feature extraction were performed via 3D Slicer (version 4.11.0; http://www.slicer.org) and its extension “slicer radiomics” derived from Pyradiomics. A detailed description is presented in the supplement.

### Feature selection and radiomics score building

A three-step feature selection procedure was applied to the training cohort to construct the radiomics score (RS). First, features with both inter-observer and intra-observer ICC higher than 0.75 were selected for further analysis. Second, a univariable analysis was performed using the Cox proportional hazards model. All features were ranked according to the *p* value. Only features with *p* value < 0.05 were kept for further analysis. With the remaining features, the least absolute shrinkage and selection operator (LASSO) method was applied to select features from training cohort. LASSO regression is an appropriate feature selecting method for high-dimensional data which has been applied in many radiomics studies.

A time-dependent receiver operator characteristic curve (ROC) was applied to evaluate the predictive accuracy of rad-score and calculate the best cutoff value.

### Prognostic value of radiomics score

The potential association between radiomics score and DFS was assessed in the training cohort and validation cohort. Patients were divided into a high-score group (radiomics score higher than the cutoff value) and a low-score group (radiomics score lower than cutoff value) groups according to the cutoff rad-score value. Kaplan–Meier survival analysis was performed in both cohorts. The baseline characteristics between the two groups were compared with Chi-square test.

Univariate and multivariate Cox proportional hazards model was applied in this study. Factors with a *p* value < 0.1 in univariate analysis and clinically significant variants were included in multivariate analysis.

### External validation of radiomics score

The feasibility of the radiomics score was further validated using the TCGA-BRCA cohort from the TCIA (external validation). The same radiomics score calculation procedure was applied, and the same cutoff value was used to divide the cohort into the low- and high-score groups.

### Predictive value of radiomics score

We constructed three prediction models to assess the incremental prognostic value of the radiomics score. The TNM model was based on cTNM staging, and the clinical model was based on all clinicopathological data, including the TNM stage, ER status, and PR status and subtype. Eventually, the radiomics model was based on radiomics and clinicopathological data. The concordance index (C-index) and AUC were calculated for each model.

The incremental prognostic value of the radiomics score to TNM staging was calculated using integrated discrimination improvement (IDI) and net reclassification improvement (NRI). Decision curve analysis (DCA) was performed to analyze the clinical usefulness of the radiomics model, which incorporated radiomics score and clinicopathological data via quantitatively measuring the net benefit at different threshold probabilities.

We further explored the prognostic value of the radiomics score in the non-pCR subgroup and the HR + /HR − subgroup. Subgroup analysis was performed in the training and validation cohorts. Kaplan–Meier survival analysis and multivariate analysis were applied.

### Development and validation of radiomics nomogram

A radiomics nomogram was constructed based on the radiomics model. The calibration curve and time-dependent ROC were applied to assess the calibration of the nomogram.

### Gene set enrichment analysis of groups with different radiomics scores

To examine the hypothesis that radiomics may reflect tumor heterogeneity, and to identify the association between radiomics score and gene expression, we extracted and incorporated genomic data with the radiomics score in the TCGA-BRCA cohort. Patients from the TCGA-BRCA cohort with RNA-seq and microRNA-seq were enrolled (*n* = 64 cases). RNA-seq data were also downloaded from the TCGA. Patients were divided into three groups according to the radiomics score. Patients with high radiomics scores (top 1/3; 21 cases) were defined as the high-score group, patients with low radiomics scores (bottom 1/3; 21 cases) were defined as the low-score group, and patients who did not fall into either category were defined as the intermediate-score group (22 cases).

Gene set enrichment analysis (GSEA) was performed to identify enriched biological pathways associated with the high- and low-score groups. FDR < 0.1 and *p* < 0.05 were considered statistically significant. False discovery rate was calculated using the Benjamini and Hochberg procedure. The gene expression data of more than 100 normal breast tissues in the TCGA were set as the baseline.

### Identification of differentially expressed genes (DEGs) and functional annotation

DEGs (mRNA, lncRNA, and miRNA) between the high- and low-score groups were identified using “lemma” and “edgeR.” An absolute log2-fold change (|FC|) > 1 and an adjusted *p* value < 0.05 were set as the cutoff criteria. We conducted Gene Ontology (GO) enrichment, Kyoto Encyclopedia of Genes and Genomes (KEGG) pathway analyses based on the DEGs using a web tool Metascape (http://metascape.org/) and the “clusterProfiler” package. GO terms and KEGG pathways with adjusted *p* values < 0.05 were considered statistically significant and visualized.

### Prognostic value of DEGs

The identified DEGs were examined for their prognostic value. Survival analysis was performed based on TCGA-BRCA data. Data were obtained from UCSC Xena (https://xenabrowser.net/). Clinical and RNA-seq data were derived from TCGA-BRCA and GSE118527. GSE118527 (FUSCC-TNBC cohort) was from our center and applied to further validate the prognostic value of the DEGs [22].

### Identification of the association of radiomics score with the tumor microenvironment and immunophenotype

To test the hypothesis that the radiomics score could reflect the heterogeneity of the tumor microenvironment, the ESTIMATE method was used to infer tumor purity of the high- and low-score groups [23]. Furthermore, The TCGA TIL MAP was applied to assess the spatial distribution of tumor infiltration lymphocytes (TILs) in these patients. The TIL map data were downloaded from the TCIA (https://cancerimagingarchive.net/datascope/TCGA_TilMap) [24]. The CIBERSORTx web tool was applied to characterize the abundance of 22 immune cell types based on the RNA-seq data of the high- and low-score groups [25]. Only samples with *p* values < 0.05 were kept for analysis because of the high reliability of the inferred cell composition. The abundance of the 22 infiltrative immune cells was compared between the two groups using Chi-square test. Finally, to characterize the immunophenotype difference between the high- and low-score groups, the immunophenscore (IPS) was calculated for each case and compared between the two radiomics groups using the Kolmogorov–Smirnov test (https://tcia.at/tools/toolsMain) [26].

### Association between computational histopathology and radiomics

As implied by GO analysis results, we applied an open-source software QuPath to characterize computational histopathological features [27]. The whole slide images (WSIs) of patients from different score groups were obtained from https://portal.gdc.cancer.gov/.

Computational histopathological features were extracted by QuPath to represent the levels of tumor cell differentiation and tumor morphology. The features extraction procedure was as follows: ROI on WSIs was cut into tiles with the width and height set as 100 $\mathrm{\mu m}$; next, given the size difference between tumor cells and TILs within ROI, the QuPath automated cell detection function was applied to detect tumor cells. The cell detection strategy was similar to that outlined in a previous study. The detailed thresholds were as follows: detection image, hematoxylin OD; requested pixel size, 0.3 µm; background radius, 8 µm; median filter radius, 0 µm; sigma, 1.5 µm; minimum cell area, 24 µm^2^; maximum cell area, 1,000 µm^2^; and threshold, 0.1. The quality control of the automated cell detection was confirmed by a pathologist. A total of 85 features based on tumor cells and tumor tiles were calculated by QuPath and then aggregated across the case-level tiles by the min, median, max, 25-quantiles, and 75-quantiles of the values.

The correlation between radiomics features and computational histopathological features was assessed. Computational histopathological features were compared between the two groups (low vs intermediate, low vs high). FDR < 0.1 and *p* < 0.05 was consider statistically significant (FDR was calculated using Benjaminiand Hochberg procedure).

### Statistical analysis

The differences in baseline characteristics were examined using two-sample *t* test, Pearson’s Chi-square test, and Fisher’s exact test as appropriate. The Kaplan–Meier method and log-rank test were used to estimate DFS. Multivariate analysis was conducted using the Cox proportional hazards model. The C-index was applied to measure the accuracy of the predictive model. The cell type distribution was assessed by Chi-square test, and the proportion of immune cells was assessed by t-test.

Statistical analysis was performed using R software (version 4.0.2, www.Rproject.org) and SPSS (Chicago, v20). A two-sided *p* value < 0.05 was considered to be statistically significant.

## Results

### Baseline characteristics

The study overview is presented in Fig. 1. The baseline characteristics in the training cohort and validation cohort are presented in Table 1. The two cohorts were well-balanced (*p* values ranging from 0.200 to 0.971). The median follow-up time for all 278 patients was 52 months. In the training cohort, the 5-year DFS and OS were 76.0% (95% confidence interval [CI] 68.2–83.8%) and 89.6% (95% CI 84.1–95.1%), respectively. In the validation cohort, the 5-year DFS and OS were 72.3% (95% CI 64.3–80.3%) and 85.7% (95% CI 79.2–92.2%), respectively. As for the final follow-up, the number of primary endpoint events was 32 (23.0%) and 38 (27.3%) for the training and validation cohorts, respectively.

**Table 1:** Demographic and clinical characteristics

	Training cohort	Test cohort	
Characteristics	Number (%)	Number (%)	*P* value
Case	139 (100.0%)	139 (100.0%)	
*Age (y)*			0.628
≤ 45	58 (41.7%)	62 (44.6%)	
≥ 46	81 (58.3%)	77 (55.4%)	
*cT stage*			0.521
T1	15 (10.8%)	11 (7.9%)	
T2	83 (59.7%)	92 (66.2%)	
T3	27 (19.4%)	20 (14.4%)	
T4	14 (10.1%)	16 (11.5%)	
*cN stage*			0.200
N0	19 (13.7%)	9 (6.5%)	
N1	75 (54.0%)	76 (54.7%)	
N2	27 (19.4%)	35 (25.2%)	
N3	18 (12.9%)	19 (13.7%)	
*ypT stage*			0.360
T0	52 (37.4%)	49 (35.0%)	
T1	63 (45.3%)	75 (53.6%)	
T2	20 (14.4%)	12 (8.6%)	
T3	4 (1.4%)	4 (1.4%)	
*ypT stage*			0.332
T0	64 (46.0%)	53 (38.1%)	
T1	36 (25.9%)	43 (30.9%)	
T2	24 (17.3%)	32 (23.0%)	
T3	15 (10.8%)	11 (7.9%)	
*pCR*			0.887
pCR	33 (23.7%)	32 (23.0%)	
Non-pCR	106 (76.3%)	107 (77.0%)	
*ER status*			0.708
Positive	90 (64.7%)	87 (62.6%)	
Negative	49 (35.3%)	52 (37.4%)	
*PR status*			0.400
Positive	77 (55.4%)	70 (50.4%)	
Negative	62 (44.6%)	69 (49.6%)	
*HER-2 status*			0.694
Positive	96 (69.1%)	99 (71.2%)	
Negative	43 (30.1%)	40 (28.8%)	
*Adjuvant chemotherapy*			0.462
Yes	81 (58.3%)	87 (62.6%)	
No	58 (41.7%)	52 (37.4%)	
*IMNI*			0.407
Yes	32 (23.0%)	38 (27.3%)	
No	107 (77.0%)	101 (72.7%)	
*Anti-HER2 therapy*			0.971
Yes	11 (15.7%)	11 (15.9%)	
No	59 (84.3%)	58 (84.1%)	
*Hormonal therapy*			0.622
Yes	32 (23.0%)	32 (23.0%)	
No	102 (73.4%)	105 (75.5%)	
Unknown	5 (3.6%)	2 (1.4%)	

*ER* estrogen receptor, *PR* progesterone receptor, *HER2* human epidermal growth factor receptor 2, *pCR* pathologic complete response, *NAC* neoadjuvant chemotherapy, *IMNI* internal mammary node irradiation

### Building the radiomics score and predictive model

#### Feature selection and radiomics score building

The detailed workflow of the radiomics model construction and clinical value assessment is shown in Additional file 1: Fig. S1. Among the 850 features extracted from the training cohort, 616 features with inter- and intra-ICC (intraclass correlation coefficient) > 0.75 were selected (Additional file 2: Fig. S2A, B), then the univariate Cox model identified 250 features with *p* < 0.05 among the 616 features. A further 15 features with nonzero coefficients were selected from the 250 features using the LASSO (Additional file 2: Fig. S2C, D), and the radiomics score was then calculated accordingly. The formula is shown in the supplement. The cutoff value was − 51.7682 calculated by time-dependent ROC. The AUC was 0.891 (Additional file 2: Fig. S2E). Accordingly, patients from both the training and validation cohorts were classified into the high-score group (radiomics score >  − 51.7682) and low-score group (radiomics score ≤  − 51.7682).

#### Analysis of the prognostic value of the radiomics score

**Fig. 2:**
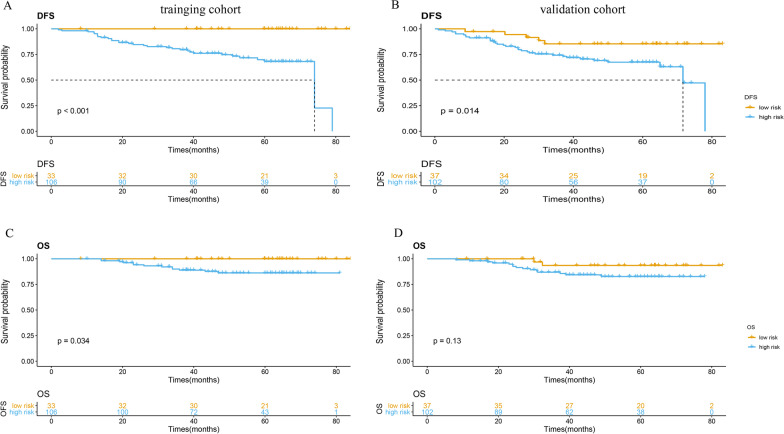
Kaplan–Meier survival analysis according to the best cutoff value of the radiomics score in the training cohort (left pane) and validation cohort (right pane). We calculated *p* values using the log-rank test. **a**, **b** Disease-free survival analysis. **c**, **d** Overall survival analysis

Patients in the low-score group showed a significantly better DFS and OS compared to those in the high-score group in the training cohort (*p* < 0.001, *p* = 0.034, respectively) (Fig. 2a, b). The median DFS was 74.0 months (95% CI 62.8–82.3) in the high-score group, whereas the median DFS was not reached in the low-score group. The results were consistent in the validation cohort, in that patients in the low-score group showed a significantly better DFS (*p* = 0.014) (Fig. 2c, d).

Clinicopathological factors, including age, cTNM stage, HR status, HER2 status, pCR, IMNI, and radiomics score, were included in the multivariate model. Multivariate analysis showed that the radiomics score was an independent prognostic factor for DFS in the training cohort (*p* < 0.001, HR 3.866, 95% CI 2.537–5.891) (Table 2), which was similar to the result observed in the validation cohort (*p* = 0.042, HR 1.002; 95% CI 1.000–1.005) (Table 3).

**Table 2:** Multivariable analysis for DFS in the training cohort

Variables	HR	95% CI	*p* value
Age	1.0431	1.006–1.082	0.024
cTNM stage			
IIIa-b vs II	1.6461	0.685–3.957	0.265
IIIc vs II	1.8162	0.622–5.302	0.275
pCR (Yes vs No)	1.229	0.4647–3.248	0.678
IMNI (Yes vs No)	0.724	0.284–1.845	0.498
HR (HR + vs HR −)	0.742	0.301–1.829	0.516
HER2 (HER2 + vs HER2 −)	0.688	0.285–1.664	0.407
Radiomics score	3.866	2.537–5.891	< 0.001

*HR* hormonal receptor, *HER2* human epidermal growth factor receptor 2, *IMNI* internal mammary node irradiation

**Table 3:** Multivariable analysis for DFS in the validation cohort

Variables	HR	95% CI	*p* value
Age	0.981	0.951–1.012	0.232
cTNM stage (III vs II)			
IIIa-b vs II	1.092	0.510–2.339	0.820
IIIc vs II	1.884	0.752–4.717	0.176
pCR (Yes vs No)	1.022	0.456–2.295	0.957
IMNI (Yes vs No)	1.427	0.702–2.904	0.326
HR (HR + vs HR −)	1.297	0.553–3.041	0.550
HER2 (HER2 + vs HER2 −)	1.085	0.495–2.380	0.839
Radiomics score	1.002	1.000–1.005	0.042

*HR* hormonal receptor, *HER2* human epidermal growth factor receptor 2, *IMNI* internal mammary node irradiation

#### External validation of the radiomics score

The TCGA-BRCA cohort consisting of 91 cases served as an external validation. The same feature extraction procedure and radiomics score calculation function were applied. The cutoff value was − 51.7682; patients in this cohort were further divided into the high-score group and low-score group. Survival analysis showed a similar result, in that the high-score group was associated with a poor prognosis (*p* = 0.041), which validated the radiomics score (Additional file 3: Fig. S3A).

#### Development and validation of the radiomics nomogram

**Fig. 3:**
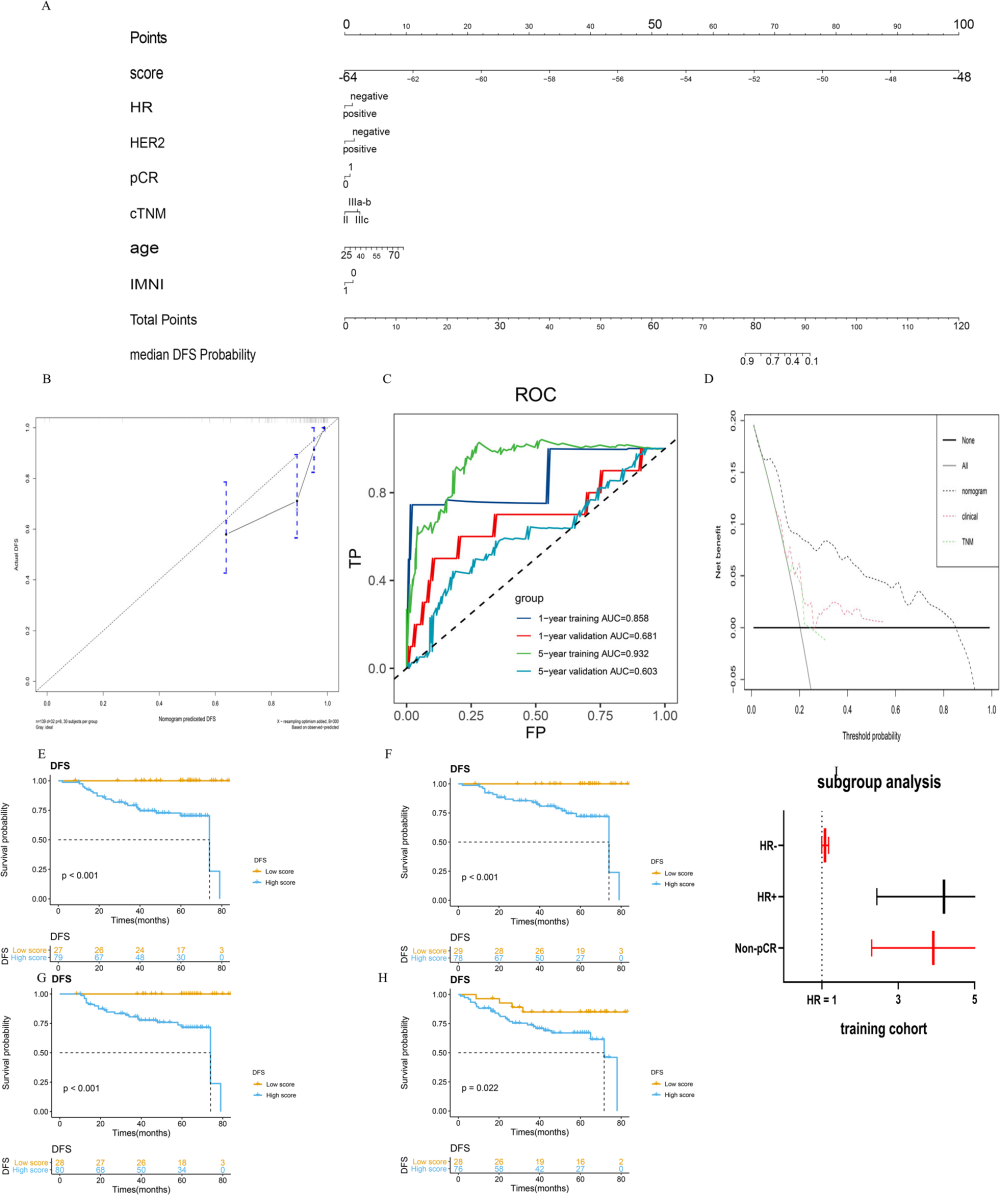
Predictive performance of the radiomics score. We constructed and validated a radiomics-based model. Subgroup analysis was performed. **a** A radiomics-based nomogram based on multivariate analysis to estimate the DFS of LABC patients. **b** Calibration curves of the radiomics nomogram showed good agreement between the estimated and actual survival time. **c** Time-dependent ROC showed good predictive accuracy at 1,5-year timepoint in both cohorts. **d** DCA analysis was performed in all three models and showed that radiomics-based nomogram outperformed the TNM model and clinical model. **e**–**i** Kaplan–Meier survival analysis was in non-pCR and HR-positive subgroups in both cohorts. Radiomics score stratified tumor recurrence risk in non-pCR subgroup and HR-positive subgroup. Forest plot was shown based on training cohort

As previously described, the radiomics features extracted in this study showed a clear association with breast cancer prognosis. We further used the radiomics score to build a prognostic model. A radiomics-based nomogram was constructed based on the multivariate analysis of the training cohort (Fig. 3a). The C-index for the radiomics nomogram for the training and validation cohorts was 0.820 (95% CI 0.744–0.896) and 0.612 (95% CI 0.528–0.696), respectively. The C-index of radiomics score alone was 0.810 (95% CI 0.743–0.877) and 0.614 (95% CI 0.522–0.706) for training cohort and validation cohort. The C-index for the cTNM-based nomogram for the training and validation cohorts was 0.620 (95% CI 0.504–0.700) and 0.516 (95% CI 0.426–0.606), respectively. The calibration curve plot showed good agreement between observation and prediction in the training and validation cohorts (Fig. 3b, Additional file 4: Fig. S4). The time-dependent ROC curve at two timepoints showed good predictive accuracy (Fig. 3c).

#### The predictive value of radiomics score

The prognostic model of TNM was first constructed based on cTNM staging only. IDI and NRI were used to evaluate the incremental predictive value of the radiomics score to cTNM staging. The inclusion of the radiomics score in the TNM model yielded an IDI of 0.247 (95% CI 0.126–0.343, *p* < 0.01) and 0.027 (95% CI 0.002–0.058, *p* < 0.01) in the training and validation cohorts, respectively. The NRI was 0.539 (95% CI 0.244–0.682, *p* < 0.01) and 0.127 (95% CI 0.029–0.346, *p* = 0.07) in the training and validation cohorts, respectively. We also constructed a clinical model based on all clinicopathological parameters except radiomics score. DCA showed that the radiomics model outperformed the TNM model and clinical model (Fig. 3d).

#### Subgroup analysis

Non-pCR patients require additional systemic therapy. Due to the limited number of pCR patients (65 cases, 23.4%), subgroup analysis was performed on the non-pCR subgroup only. The results showed that radiomics scores could stratify tumor recurrence risk in the non-PCR subgroup (*p* < 0.001) (Fig. 3e, f). Consistent with the non-pCR subgroup results, the radiomics score also stratified tumor recurrence risk in the HR + subgroup (*p* < 0.001 for training cohort, *p* = 0.022 for validation cohort) (Fig. 3g, i). Multivariate analysis showed that the radiomics score was an independent prognostic factor (data shown in supplement).

#### Gene set enrichment analysis of groups with different radiomics scores

**Fig. 4:**
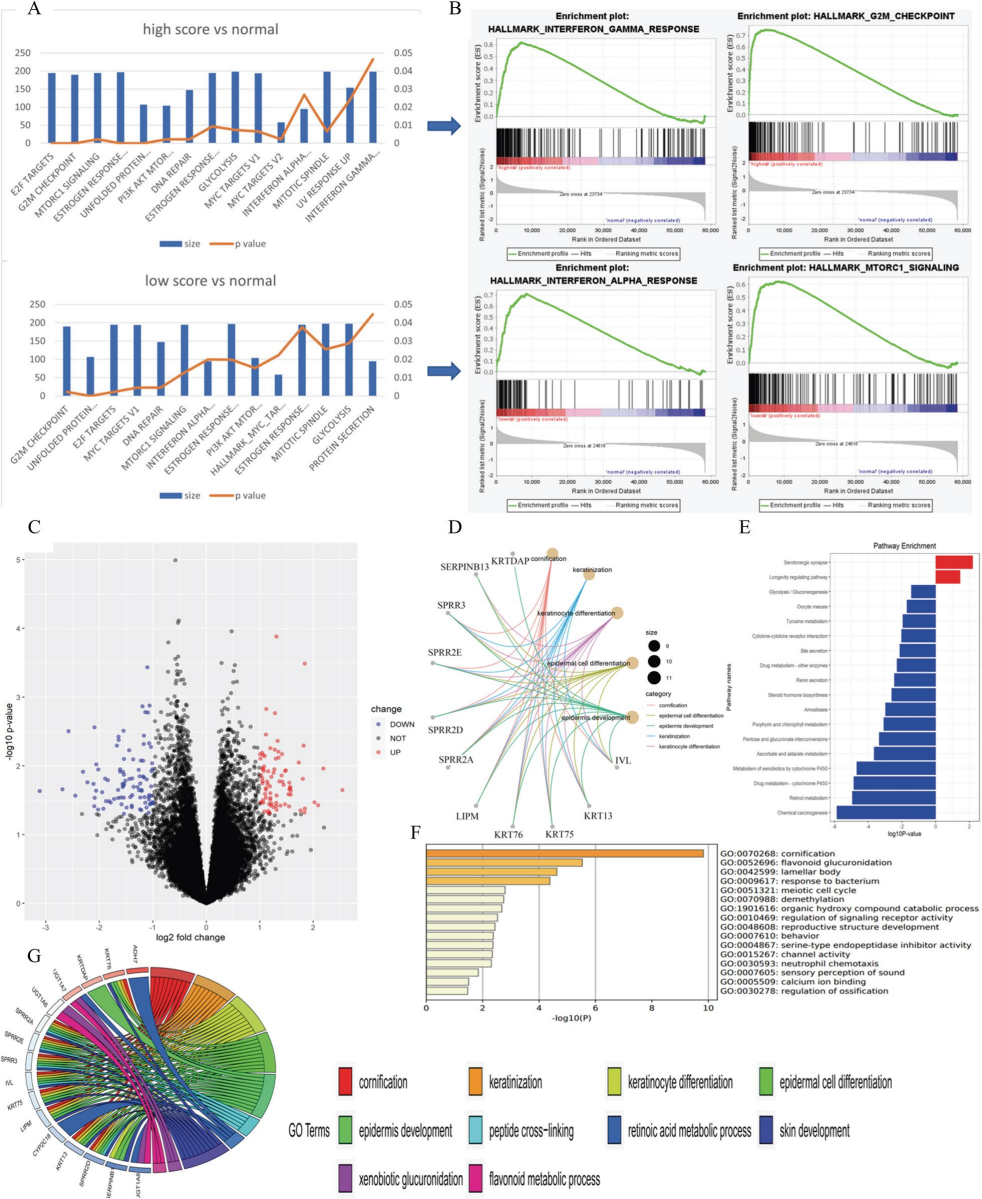
The association between radiomics score and gene expression pattern. **a**, **b** GSEA analysis in low-score group and high-score group. Hallmark gene sets with *p* value < 0.05 were presented. Estrogen response pathway, DNA repair pathway, mTOR pathway, and interferon pathway were enriched. **c** Volcano plot showed upregulated and downregulated genes in low-score group and high-score group. Over 170 DEGs were identified. **d**–**g** function annotation was performed in DEGs. GO analysis showed that DEGs were relate to cornification, keratinization, and keratinocyte differentiation, suggesting that low-score group were different from high-score group in terms of cell differentiation

Researchers have hypothesized that radiomics reflects tumor heterogeneity. We supposed that radiomics scores may reflect tumor heterogeneity by showing an association with gene expression. We purposed that the gene expression pattern existed in the different radiomics score group. Thus, the association between radiomics score and gene expression was investigated, and the workflow is presented in Additional file 5: Fig. S5. Patients from the TCGA-BRCA cohort were equally divided into high-score, intermediate-score, and low-score subgroups based on individual radiomics score. GSEA showed that DNA repair, G2/M checkpoint, and PI3K/Akt/mTOR pathways were enriched in both the high- and low-score groups, which is consistent with the findings of a previous study [15] (Fig. 4a). Interestingly, immune-related pathways were also enriched. Interferon-α pathway was enriched in both groups, and the interferon-γ pathway was enriched only in the high-score group compared to normal samples (Fig. 4b).

#### Identification of DEGs and functional annotation

DEGs between the high- and low-score groups were calculated. A total of 174 DEGs were identified using RNA-seq from the TCGA, among which 93 DEGs were upregulated and 81 DEGs were downregulated in the low-score group (Fig. 4c).

Functional annotation was applied to differentially expressed protein-coding genes using both KEGG pathway and GO analysis (Fig. 4d, e). Cell metabolism pathways and cytokine–cytokine interaction pathways were downregulated, while GO analysis showed that biological processes, such as cornification, epidermal cell differentiation, and cell metabolism, were enriched (Fig. 4f, g). These results indicated the tumors in the low-score group were different from those in the high-score group in terms of cell differentiation.

**Fig. 5:**
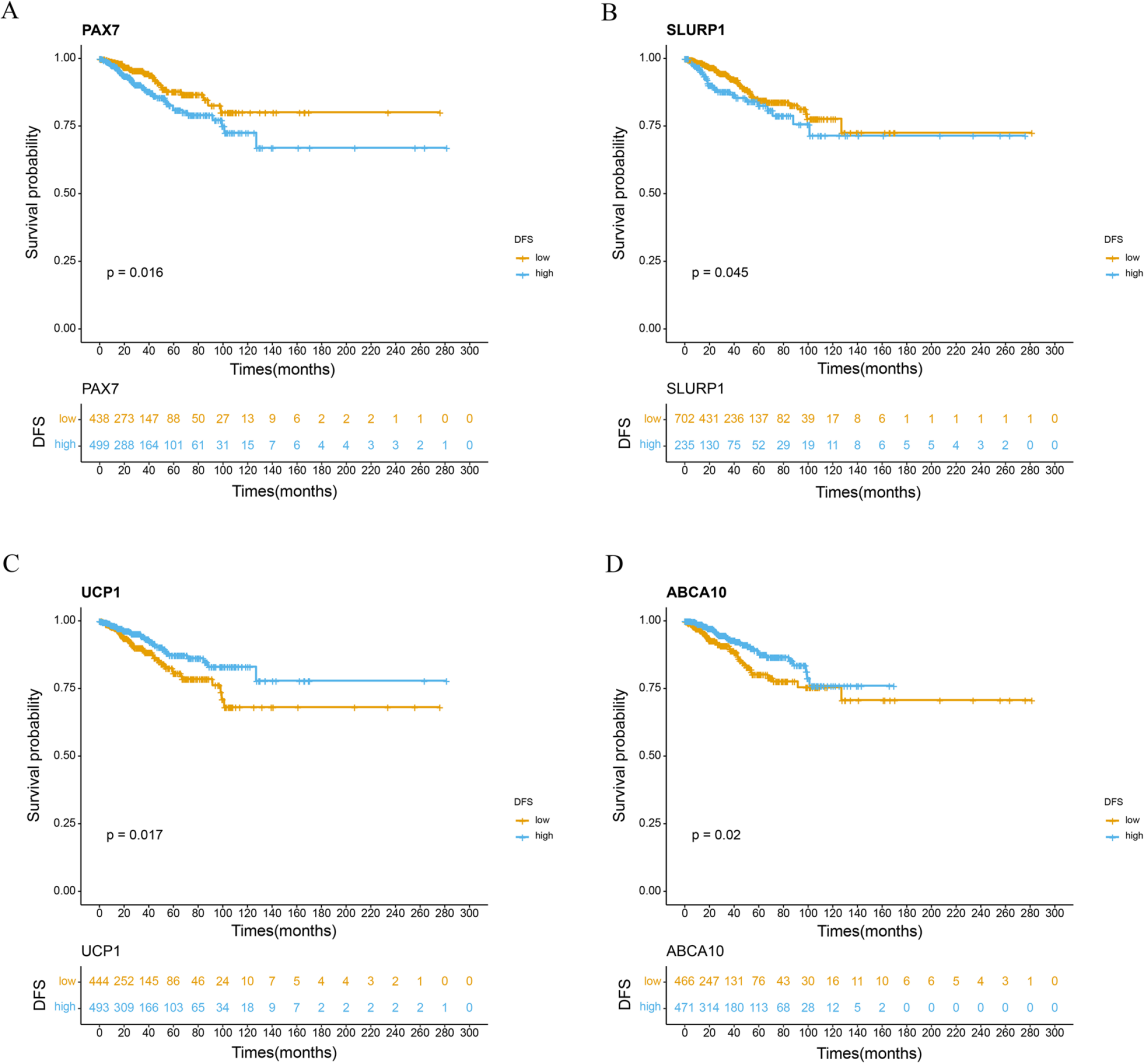
Kaplan–Meier survival analysis according to the optimal or median expression level of 4 DEGs. We calculated *p* values using the log-rank test. **a**–**d** DFS analysis of PAX7, SLURP1, UCP1 and ABCA10 based on public cohort (TCGA-BRCA)

As addressed previously, the high-score group was associated with poor prognosis, and the low-score group was associated with good prognosis. Therefore, we supposed that the upregulated DEGs in the low-score group may be associated with a good prognosis and that the downregulated DEGs in the low-score group may be related to poor prognosis; thus, to understand this further, survival analysis was applied to these DEGs. The results demonstrated that DEGs downregulated in the low-score group, such as skin differentiation marker SLURP1 and transcription factor PAX7, were indeed associated with poor prognosis (*p* = 0.045, *p* = 0.016, respectively), while those upregulated in the low-score group, such as UCP1 and ABCA10, were associated with good prognosis (*p* = 0.017, *p* = 0.020, respectively) (Fig. 5a, b). We found that most (24/28, 85.7%) DEGs fitted our assumption (Fig. 5a–d, Additional file 6: Fig. S6, Additional file 11: Table S1).

#### Association of radiomics score with the tumor microenvironment and immunophenotype

As shown previously, immune-related pathways, such interferon-α pathway and interferon-γ pathway, were enriched in the low- and high-score groups by GSEA, and cytokine–cytokine interaction pathways were downregulated in the low-score group. These results suggest that the tumor immunity and tumor microenvironment are different between the low- and high-score groups. Therefore, we investigated the association between radiomics and heterogeneity of the tumor microenvironment.

**Fig. 6:**
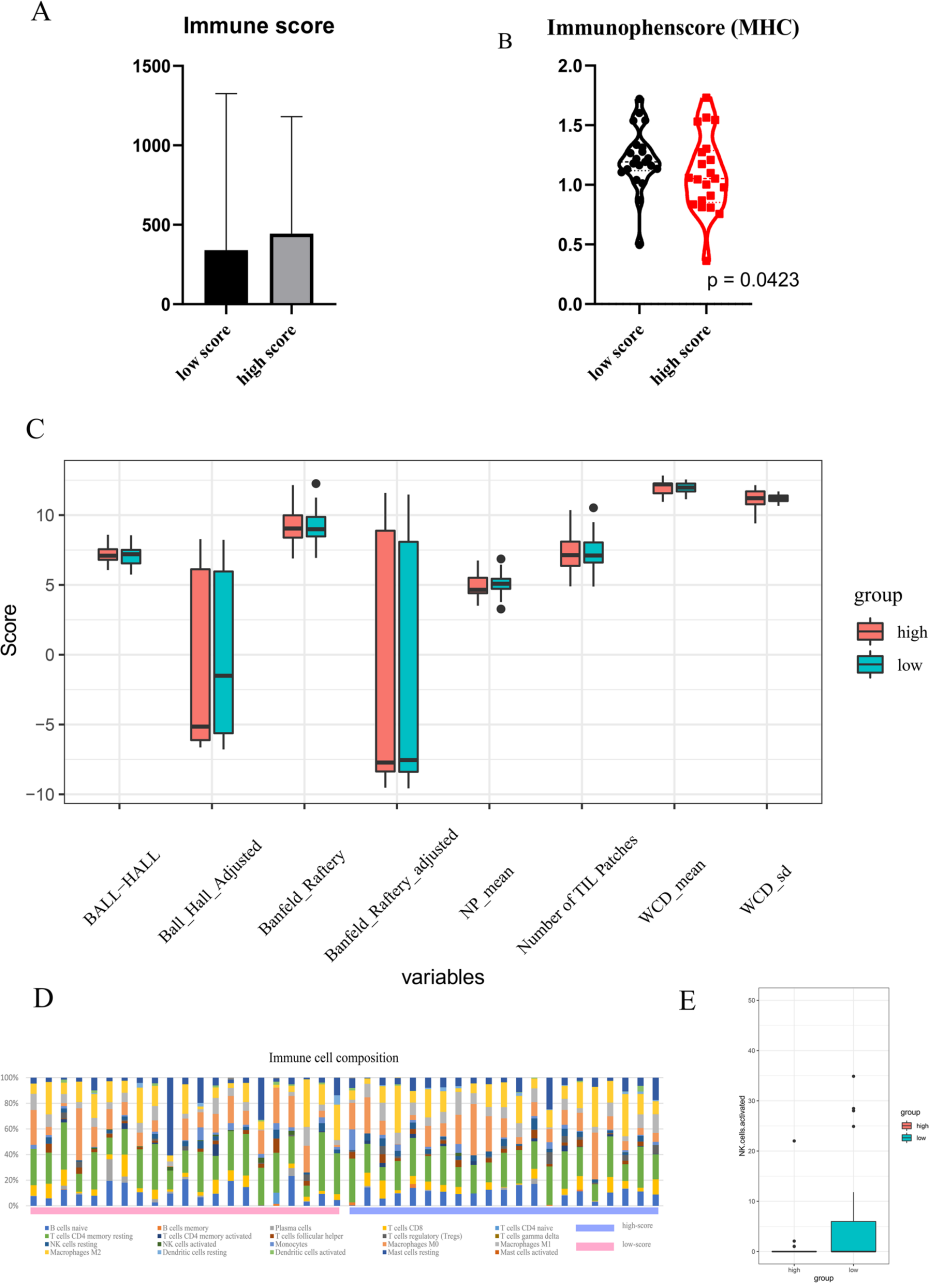
The association between radiomics score and tumor microenvironment. **a** The immune score calculated by ESTIMATE showed that tumor purity were not significantly different between two groups. **b** MHC score calculated by tumor immunophenscore were significantly lower in high-score group, indicating a better change to avoid T cell recognition. **c** TILs map showed no significant difference in the spatial distribution of tumor infiltration cells between high-score group and low-score group. Five TILs map variables were presented. **d** The proportion of 22 immune cell types predicted by CIBERSORTx was significantly different in low-score group and high-score group (Chi-square test *p* value < 0.001). **e** Activated NK cell was higher in low-score group (*p* = 0.047) (**e**)

First, tumor purity was calculated using the ESTIMATE method, which showed no significant difference between the two groups (Fig. 6a); this suggested that the number of immune and stromal cells was similar between the two groups. Second, tumor immunophenotype, comprising molecules involved in tumor escape mechanisms, was assessed. The high-score group showed a significantly lower MHC molecule score, indicating the ability to avoid T cell recognition (Fig. 6b, Additional file 7: Fig. S7A–C). Third, data from TILs Map were used to assess the spatial distribution of TILs in both groups, although no significant differences were observed (Fig. 6c). Finally, given that the amount and spatial distribution of immune cells were not significantly different between groups, CIBERSORTx was applied to evaluate immune cell composition in the tumor environment. We calculated the proportion of 22 immune cell types of groups (Fig. 6d). Activated NK cell was higher in low-score group (*p* = 0.047) (Fig. 6e).

#### Association between computational histopathology and radiomics

**Fig. 7:**
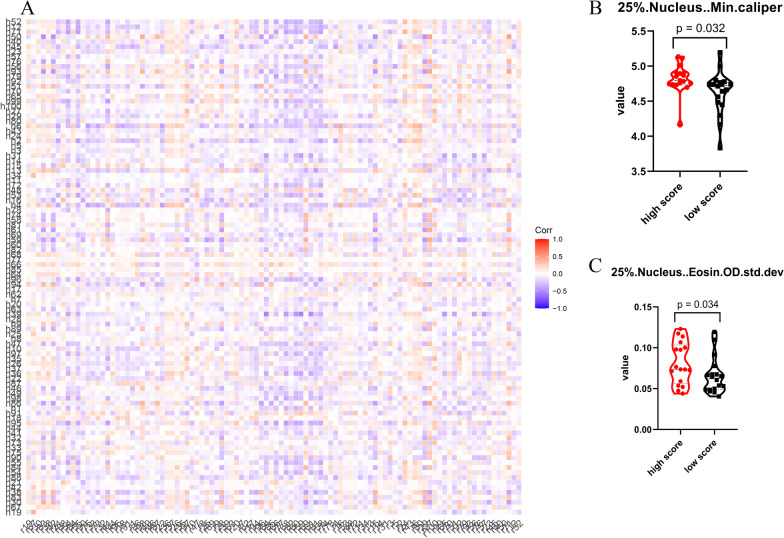
Computational histology reveals the morphological difference of tumor cells between low-score group and high-score group. **a** The correlation heatmap showed the association between radiomics features and computational histological features. **b**, **c** Tumor cells from different groups showed distinct histological features. Two histopathological features were presented

A previous study suggested that tumor cells from different imaging subtypes present with variations in histopathological features [20]. Results from functional annotation indicated that tumor cells from different score groups went through different stages of epidermal cell differentiation. Therefore, tumor cells in the low-score group may display a less “squamous cell/keratocyte” phenotype and, thus, have different morphologies. We supposed that the morphological difference resulting from cell differentiation may be subtle, so that the tumor cell phenotype would not completely transfer from adenocarcinoma to squamous carcinoma. To assess the subtle changes in tumor cell morphology, we applied computational histopathology to extract quantitative features from H.E slices of these patients. A total of 440 features were extracted for 44 patients and used in further analysis. Among the 53 patients with complete computational histopathological data, 18 cases belonged to the low-score group, 18 cases to the intermediate-score group, and 17 cases to the high-score group. We established a correlation map between computational histopathological and radiomic features (Fig. 7a, Additional file 8: Fig. S8, Additional file 9: Fig. S9, Additional file 10: Fig. S10). Twenty-three computational histopathological features were significantly different between the high- and low-score groups (Fig. 7b, c, Additional file 12: Table S2). The two groups differed in terms of cell eccentricity, nucleus caliper, and diameter. In contrast, no feature was found to be significantly different between the intermediate- and low-score groups. Thus, tumors in the high-score group were morphologically different from those in the low-score group, which supports the idea that the radiomics score reflects differences in cell differentiation.

## Discussion

To the best of our knowledge, this is the first report to predict individual DFS in patients with breast cancer who received NAC and PORT by radiomics. In this study, we constructed and validated the radiomics score to predict the DFS among patients with breast cancer after neoadjuvant chemotherapy and postoperative radiotherapy. Furthermore, by incorporating multi-omics data, we examined the hypothesis of radiomics and revealed the heterogeneity of tumor cells and the tumor microenvironment between different radiomics score groups. These results demonstrated that radiomics could predict the prognosis of patients with locally advanced breast cancer and reveal the heterogeneity of tumor cells and the tumor microenvironment.

Radiomics is a noninvasive approach that extracts quantitative features from medical imaging and reflects tumor heterogeneity. Compared to other conventional methods such as biopsy, radiomics is low-cost, safe, and repeatable and can be used to evaluate tumor heterogeneity at any time point. Therefore, radiomics have been applied in many aspects of breast cancer research, such as tumor staging and prognosis prediction [28, 29]. The estimation of DFS is important for individualized management of breast cancer patients. Only a few studies have provided insights into breast cancer prognosis using radiomics. Park et al. reported that radiomics could predict the DFS of patients with breast cancer [11]. They selected four radiomic features using LASSO, which was the same feature selection method used in our study. Multivariate analysis demonstrated that a high radiomics score was associated with poor DFS. Wu et al. reported that radiomic features could stratify patients with breast cancer based on recurrence-free survival (RFS) [15].

However, few studies have examined the prognostic value of radiomics in DFS estimation for LABC, and most previous studies have focused on early-stage breast cancer. For example, more than 40% patients with breast cancer were stage I, and patients after NAC were excluded from the study reported by Park et al. Moreover, given that not all of the enrolled patients received RT in these two studies, the evidence supporting the application of radiomics in the estimation of prognosis in patients with NAC and PORT is limited. The assessment of prognosis for patients with breast cancer after NAC and PORT is of great importance for individualized patient management. Therefore, we constructed this study and validated a radiomics score based on 15 radiomic features selected from 851 features to assess the DFS for patients with locally advanced breast cancer after NAC and PORT. We also constructed a nomogram integrating clinicopathological factors with radiomics score, which showed good predictive accuracy. Additionally, we assessed the incremental predictive value of the radiomics score and demonstrated that incorporating the radiomics score could improve the predictive performance of the TNM staging system. The results of DCA showed that incorporating the radiomics score to the TNM staging system was superior to the existing TNM staging system in terms of predictive accuracy.

Previous studies have addressed the relationship between pCR and survival outcome [30]. Results from the CREATE-X trial supported that additional consolidate treatment improved the survival outcome in non-pCR patients [31]. However, we divided non-pCR patients into two groups based on radiomics score and found that low radiomics score was associated with a better prognosis in non-pCR patients. This finding demonstrated that the radiomics score could be used to further tailor subsequent treatment. Radiomics score allows precise selection of patients who need treatment or have a chance to omit additional treatment.

After constructing and validating a radiomics score, we examined the theoretical basis: whether and how radiomics reflect tumor heterogeneity. Previous research has identified gene expression patterns in different image subtypes. Hugo et al. identified the association between gene expression and radiomic features in lung cancer and head & neck squamous carcinoma. Wu et al. and Fan et al. identified pathways enriched in breast cancer radiomic features, including DNA repair and proliferation [15, 16]. Consistent with the GESA of our study, Wu et al. found that pathways such as mTOR were associated with image subtype, while cell metabolism pathways were also enriched in the low-score group.

Notably, the interferon pathway and cytokine–cytokine interaction pathway were also enriched in our study, which suggested that the tumor immunity and tumor microenvironment were different in the high- and low-score groups, and further hinted that radiomics reflects heterogeneity derived from the tumor microenvironment. Yu et al. reported that the TIL level may be related to a few radiomics features [32]. Therefore, we investigated the tumor microenvironment from three angles: (1) tumor purity (the amount of tumor stroma and TILs); (2) the spatial distribution of TILs; and (3) the proportion of different types of immune cells. We found that the proportion of immune cells was significantly different between image subtypes. Notably, NK cell was higher in the low-score group. NK cells are important and promising targets for cancer immunotherapy, and tumors with high NK cell levels may benefit from NK cell therapy. Additionally, a previous study constructed an immunophenotype score based on three classes of molecules involved in tumor immune escape mechanism. We found that the high-score group had lower MHC molecule expression, suggesting that the poor prognosis of the high-score group was caused by the escape from tumor surveillance and T cell recognition.

Interestingly, GO analysis demonstrated that genes related to cornification, keratinization, and epidermal cell differentiation were enriched in downregulated DEGs, suggesting that tumors of the high-score group underwent epidermal cell differentiation and were different from those of the low-score group in terms of degree of differentiation. Additionally, the marker for epidermal cells was significantly higher in the high-score group.

H.E slices are commonly used to determine cell type and differentiation degree [20, 33]. Previous studies showed that, like radiomics, computational histopathology could reflect particular gene expression patterns and mutation by extracting features from WSI. Shao et al. reported that the combination of radiomics and computational histopathology could predict survival outcome [34]. Therefore, we applied computational histopathology to quantify tumor morphology in different MRI-based image subtypes. We found that tumors of the high-score group were morphologically different from those of the low-score group, which indicated that the tumor heterogeneity revealed by radiomics could also be detected by computational histopathology. The link between radiomics and computational histopathology suggested that radiomics and computational histopathology reflected tumor heterogeneity only on different dimensions and, thus, were somewhat equivalent. Therefore, we propose a new hypothesis that tumor heterogeneity reflected by radiomics results from tumor histomorphology that stems from cell differentiation.

This study has several limitations. First, this was a retrospective analysis, and, although an independent external validation cohort was applied in this study, a multi-center prospective study with a larger data set is warranted. Second, the external validation cohort was from the TCGA. Only a small number of patients had both MRI, H.E slices, and genomic data. Although we used RNA-seq and clinical data from our center (FUSCC-TNBC), they were triple-negative breast cancer patients, while many cases in the TCGA-BRCA cohort were hormonal receptor positive. Third, radiomics features were extracted from enhanced MRI from our center, which were, to some extent, different from the MRI images in the TCIA; therefore, a consistent MRI image acquisition method is needed in the future.

## Conclusions

In conclusion, this is the first study to apply radiomics to assess survival outcome for LABC patients after NAC and postoperative RT. We present a radiomics-based prognostic tool, which effectively predicts prognosis. Radiomics may reflect differences in tumor cell differentiation and cell composition in the tumor microenvironment and hold great potential for improving individualized DFS estimations and guiding treatment strategies for patients with breast cancer.

## Supplementary Information

**Additional file 1: Fig. S1**. The workflow of radiomics score calculation and clinical application. Tumor MRI scans were segmented manually. Radiomics features were then extracted and filtered according to ICC. LASSO was applied to the selected features.

**Additional file 2: Fig. S2**. The process of the calculation of radiomics score. A, B) Univariate analysis of all radiomics features. Only features with *p* < 0.05 and ICC > 0.75 were selected. C, D) LASSO was applied to select features. A radiomics score was generated by linear combination of selected features. E) A time-dependent ROC was plotted. The best cut value was set according to the Youden index.

**Additional file 3: Fig. S3**. The prognostic value of radiomics score in the external validation cohort and the results of unsupervised clustering. A) The Kaplan–Meier analysis showed that higher radiomics score was associated with worse DFS in external validation cohort.

**Additional file 4: Fig. S4**. Calibration plot of validation cohort.

**Additional file 5: Fig. S5**. The workflow of the association between tumor heterogeneity and radiomics. Heterogeneity which stems from tumor cell and microenvironment is evaluated.

**Additional file 6: Fig. S6**. Heatmap and volcano plot of DEGs between high- and low-score groups. A-B) DEGs of miRNA. C-D) DEGs of lncRNA. E) heatmap of DEGs of mRNA. F) function annotation of downregulated DEGs.

**Additional file 7: Fig. S7**. Immunophenscore of high- and low-score group. No significant difference found between groups. A) Immunophenscore (EC) of high- and low-score group. B) Immunophenscore (CP) of high- and low-score group. C) Immunophenscore (SC) of high- and low-score group.

**Additional file 8: Fig. S8**. The radiomics features extracted in this study using “slicer radiomics”.

**Additional file 9: Fig. S9**. The computational histopathological features in this study.

**Additional file 10: Fig. S10**. The illustration of ROI segmentation on H.E slice.

**Additional file 11: Table S1**. Hazard ratio of DEGs significantly associated with prognosis of TCGA-BRCA. HR: hazard ratio.

**Additional file 12: Table S2**. Computational histopathological features significantly different between the high- and low-score groups.

## Data Availability

The datasets used and/or analyzed during the current study are available from the corresponding author on reasonable request.

## Abbreviations

LABC: Locally advanced breast cancer
DFS: Disease-free survival
NAC: Neoadjuvant chemotherapy
PORT: Postoperative radiotherapy
LRR: Locoregional recurrences
BCM: Breast cancer mortality
pCR: Pathological complete remission
ER: Estrogen receptor
PR: Progesterone receptor
HER-2: Human epidermal growth factor receptor-2
PACS: Picture archiving and communication system
AJCC: American Joint Committee on Cancer
ALND: Axillary lymph node dissection
RNI: Regional nodal irradiation
IMNs: Internal mammary lymph nodes
TCIA: The Cancer Imaging Archive
RFS: Recurrence-free survival
IDI: Integrated discrimination improvement
NRI: Net reclassification improvement
DCA: Decision curve analysis
GSEA: Gene set enrichment analysis
WSIs: Whole slide images

## References

[1] Sung H, Ferlay J, Siegel RL, Laversanne M, Soerjomataram I, Jemal A (2021). Global cancer statistics 2020: GLOBOCAN estimates of incidence and mortality worldwide for 36 cancers in 185 countries. CA Cancer J Clin.

[2] Tryfonidis K, Senkus E, Cardoso MJ, Cardoso F (2015). Management of locally advanced breast cancer-perspectives and future directions. Nat Rev Clin Oncol.

[3] Whelan TJ, Olivotto IA, Parulekar WR, Ackerman I, Chua BH, Nabid A (2015). Regional nodal irradiation in early-stage breast cancer. N Engl J Med.

[4] Poortmans PM, Collette S, Kirkove C, Van Limbergen E, Budach V, Struikmans H (2015). Internal mammary and medial supraclavicular irradiation in breast cancer. N Engl J Med.

[5] Lambin P, Leijenaar RTH, Deist TM, Peerlings J, de Jong EEC, van Timmeren J (2017). Radiomics: the bridge between medical imaging and personalized medicine. Nat Rev Clin Oncol.

[6] Lu CF, Hsu FT, Hsieh KL, Kao YJ, Cheng SJ, Hsu JB (2018). Machine learning-based radiomics for molecular subtyping of gliomas. Clin Cancer Res.

[7] Wu S, Zheng J, Li Y, Yu H, Shi S, Xie W (2017). A radiomics nomogram for the preoperative prediction of lymph node metastasis in bladder cancer. Clin Cancer Res.

[8] Nie K, Shi L, Chen Q, Hu X, Jabbour SK, Yue N (2016). Rectal cancer: assessment of neoadjuvant chemoradiation outcome based on radiomics of multiparametric MRI. Clin Cancer Res.

[9] Liang W, Yang P, Huang R, Xu L, Wang J, Liu W (2019). A combined nomogram model to preoperatively predict histologic grade in pancreatic neuroendocrine tumors. Clin Cancer Res.

[10] Zhang B, Tian J, Dong D, Gu D, Dong Y, Zhang L (2017). Radiomics features of multiparametric MRI as novel prognostic factors in advanced nasopharyngeal carcinoma. Clin Cancer Res.

[11] Park H, Lim Y, Ko ES, Cho HH, Lee JE, Han BK (2018). Radiomics signature on magnetic resonance imaging: association with disease-free survival in patients with invasive breast cancer. Clin Cancer Res.

[12] Xie T, Wang X, Li M, Tong T, Yu X, Zhou Z (2020). Pancreatic ductal adenocarcinoma: a radiomics nomogram outperforms clinical model and TNM staging for survival estimation after curative resection. Eur Radiol.

[13] Jiang Y, Chen C, Xie J, Wang W, Zha X, Lv W (2018). Radiomics signature of computed tomography imaging for prediction of survival and chemotherapeutic benefits in gastric cancer. EBioMedicine.

[14] Liu Z, Li Z, Qu J, Zhang R, Zhou X, Li L (2019). Radiomics of multiparametric MRI for pretreatment prediction of pathologic complete response to neoadjuvant chemotherapy in breast cancer: a multicenter study. Clin Cancer Res.

[15] Wu J, Cui Y, Sun X, Cao G, Li B, Ikeda DM (2017). Unsupervised clustering of quantitative image phenotypes reveals breast cancer subtypes with distinct prognoses and molecular pathways. Clin Cancer Res.

[16] Fan M, Xia P, Liu B, Zhang L, Wang Y, Gao X (2019). Tumour heterogeneity revealed by unsupervised decomposition of dynamic contrast-enhanced magnetic resonance imaging is associated with underlying gene expression patterns and poor survival in breast cancer patients. Breast Cancer Res.

[17] Aerts HJ, Velazquez ER, Leijenaar RT, Parmar C, Grossmann P, Carvalho S (2014). Decoding tumour phenotype by noninvasive imaging using a quantitative radiomics approach. Nat Commun.

[18] Fan M, Xia P, Clarke R, Wang Y, Li L (2020). Radiogenomic signatures reveal multiscale intratumour heterogeneity associated with biological functions and survival in breast cancer. Nat Commun.

[19] Zhan X, Cheng J, Huang Z, Han Z, Helm B, Liu X (2019). Correlation analysis of histopathology and proteogenomics data for breast cancer. Mol Cell Proteomics.

[20] Fu Y, Jung AW, Torne RV, Gonzalez S, Vöhringer H, Shmatko A (2020). Pan-cancer computational histopathology reveals mutations, tumor composition and prognosis. Nature Cancer.

[21] Luo J, Jin K, Chen X, Wang X, Yang Z, Zhang L (2019). Internal mammary node irradiation (IMNI) improves survival outcome for patients with clinical stage II–III breast cancer after preoperative systemic therapy. Int J Radiat Oncol Biol Phys.

[22] Jiang YZ, Ma D, Suo C, Shi J, Xue M, Hu X (2019). Genomic and transcriptomic landscape of triple-negative breast cancers: subtypes and treatment strategies. Cancer Cell.

[23] Yoshihara K, Shahmoradgoli M, Martínez E, Vegesna R, Kim H, Torres-Garcia W (2013). Inferring tumour purity and stromal and immune cell admixture from expression data. Nat Commun.

[24] Saltz J, Gupta R, Hou L, Kurc T, Singh P, Nguyen V (2018). Spatial organization and molecular correlation of tumor-infiltrating lymphocytes using deep learning on pathology images. Cell Rep.

[25] Steen CB, Liu CL, Alizadeh AA, Newman AM (2020). Profiling cell type abundance and expression in bulk tissues with CIBERSORTx. Methods Mol Biol.

[26] Charoentong P, Finotello F, Angelova M, Mayer C, Efremova M, Rieder D (2017). Pan-cancer immunogenomic analyses reveal genotype-immunophenotype relationships and predictors of response to checkpoint blockade. Cell Rep.

[27] Bankhead P, Loughrey MB, Fernández JA, Dombrowski Y, McArt DG, Dunne PD (2017). QuPath: open source software for digital pathology image analysis. Sci Rep.

[28] Chitalia RD, Rowland J, McDonald ES, Pantalone L, Cohen EA, Gastounioti A (2020). Imaging phenotypes of breast cancer heterogeneity in preoperative breast dynamic contrast enhanced magnetic resonance imaging (DCE-MRI) Scans predict 10-year recurrence. Clin Cancer Res.

[29] Zheng X, Yao Z, Huang Y, Yu Y, Wang Y, Liu Y (2020). Deep learning radiomics can predict axillary lymph node status in early-stage breast cancer. Nat Commun.

[30] Broglio KR, Quintana M, Foster M, Olinger M, McGlothlin A, Berry SM (2016). Association of pathologic complete response to neoadjuvant therapy in HER2-positive breast cancer with long-term outcomes: a meta-analysis. JAMA Oncol.

[31] Masuda N, Lee SJ, Ohtani S, Im YH, Lee ES, Yokota I (2017). Adjuvant capecitabine for breast cancer after preoperative chemotherapy. N Engl J Med.

[32] Yu H, Meng X, Chen H, Han X, Fan J, Gao W (2020). Correlation between mammographic radiomics features and the level of tumor-infiltrating lymphocytes in patients with triple-negative breast cancer. Front Oncol.

[33] Macon WR (2020). Computational histopathology and deep transfer learning: characterizing the molecular basis of tumor morphology. J Hematopathol.

[34] Shao L, Liu Z, Feng L, Lou X, Li Z, Zhang XY (2020). Multiparametric MRI and whole slide image-based pretreatment prediction of pathological response to neoadjuvant chemoradiotherapy in rectal cancer: a multicenter radiopathomic study. Ann Surg Oncol.

