# Supplementary figures and images for: Radiomics predicts the prognosis of patients with locally advanced breast cancer by reflecting the heterogeneity of tumor cells and the tumor microenvironment
Source: Breast Cancer Res. 2022 Mar 15;24:20. doi: 10.1186/s13058-022-01516-0 (PMC8922933; doi:10.1186/s13058-022-01516-0)

(a) Feature extraction → (b) Feature selection → (c) Model Validation → (d) Prognostic value

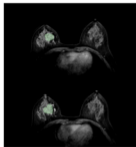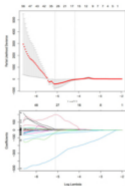

ICC results

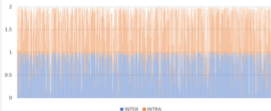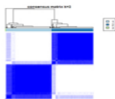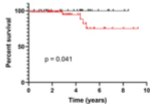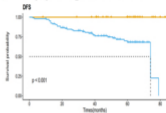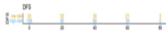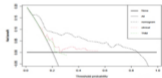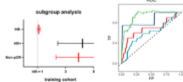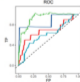

Supplement: Supplementary file 1 — Additional file 1: Fig. S1. The workflow of radiomics score calculation and clinical application. Tumor MRI scans were segmented manually. Radiomics features were then extracted and filtered according to ICC. LASSO was applied to the selected features. [file 13058_2022_1516_MOESM1_ESM.pdf]

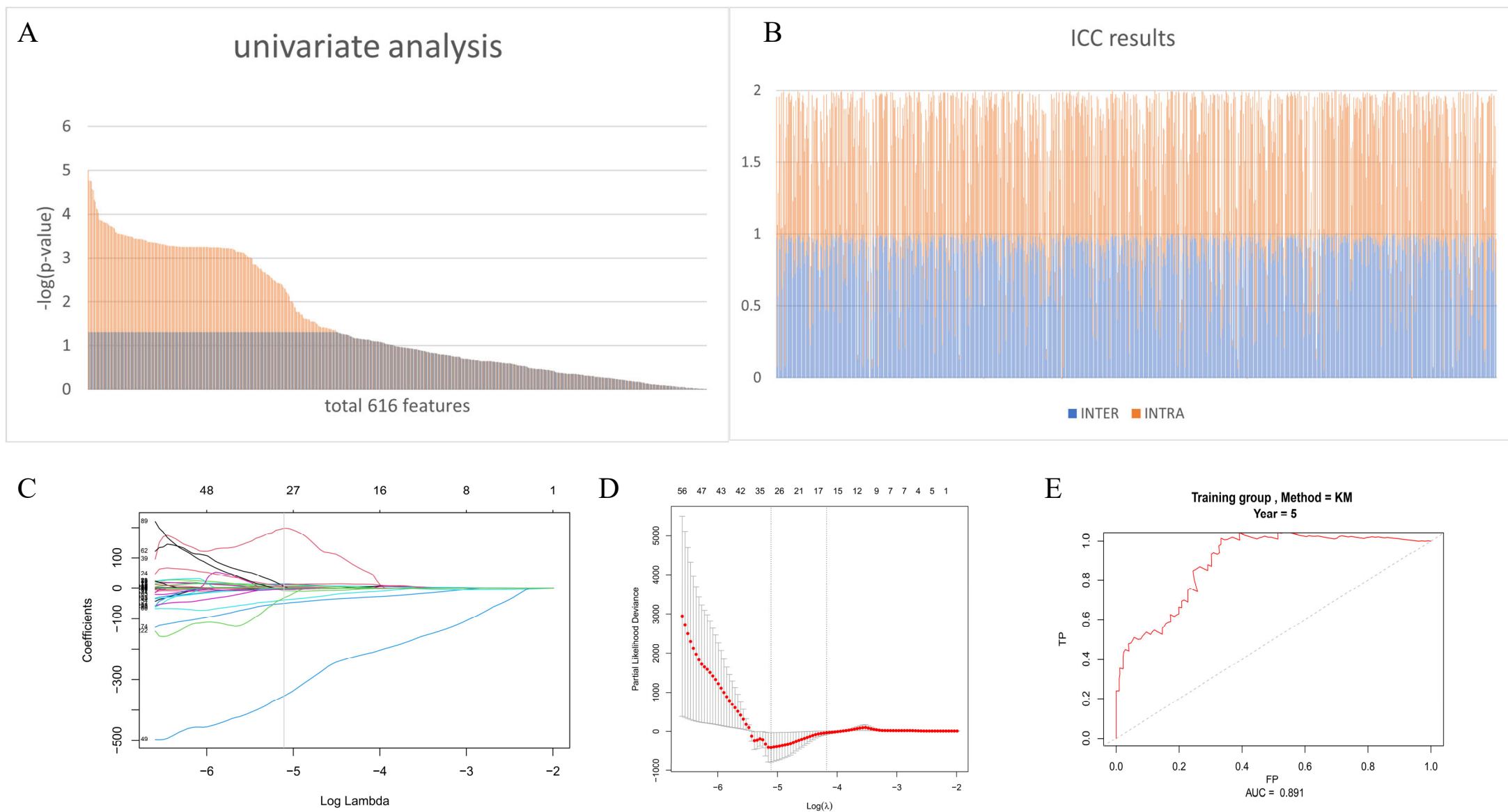

Supplement: Supplementary file 2 — Additional file 2: Fig. S2. The process of the calculation of radiomics score. A, B) Univariate analysis of all radiomics features. Only features with p < 0.05 and ICC > 0.75 were selected. C, D) LASSO was applied to select features. A radiomics score was generated by linear combination of selected features. E) A time-dependent ROC was plotted. The best cut value was set according to the Youden index. [file 13058_2022_1516_MOESM2_ESM.pdf]

A

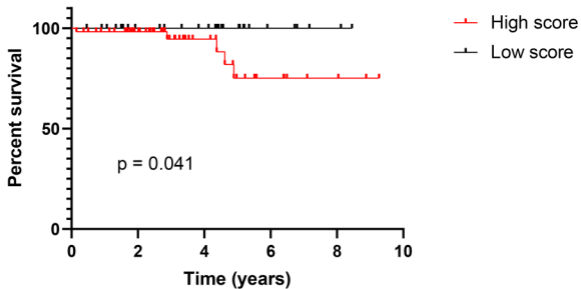

Supplement: Supplementary file 3 — Additional file 3: Fig. S3. The prognostic value of radiomics score in the external validation cohort and the results of unsupervised clustering. A) The Kaplan–Meier analysis showed that higher radiomics score was associated with worse DFS in external validation cohort. [file 13058_2022_1516_MOESM3_ESM.pdf]

# SLURP1

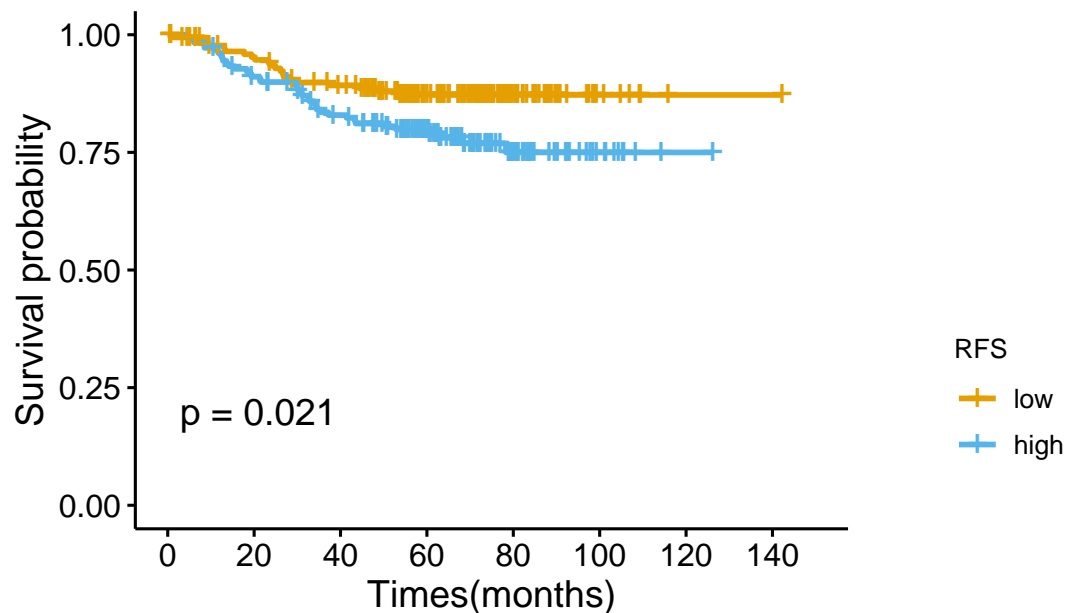

# SLURP1

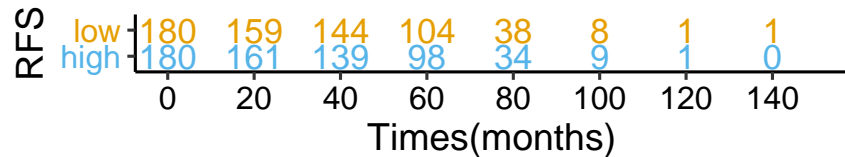

Supplement: Supplementary file 6 — Additional file 6: Fig. S6. Heatmap and volcano plot of DEGs between high- and low-score groups. A-B) DEGs of miRNA. C-D) DEGs of lncRNA. E) heatmap of DEGs of mRNA. F) function annotation of downregulated DEGs. [file 13058_2022_1516_MOESM6_ESM.pdf]

**Immunophenoscore (EC)**

**A**

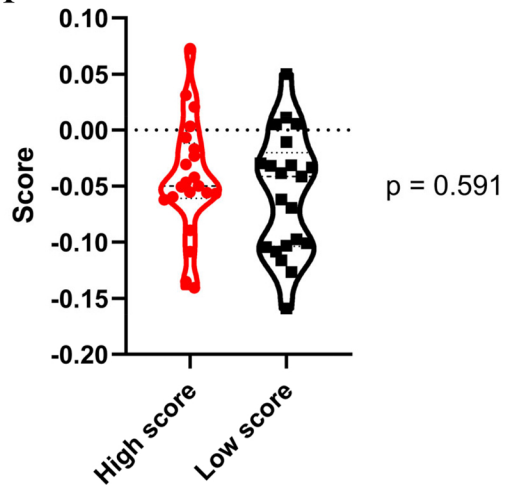

**Immunophenoscore (CP)**

**B**

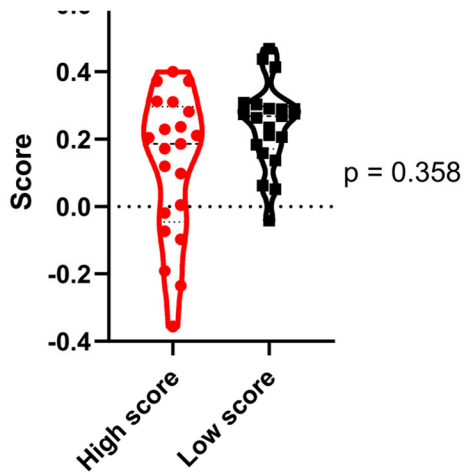

**Immunophenoscore (SC)**

**C**

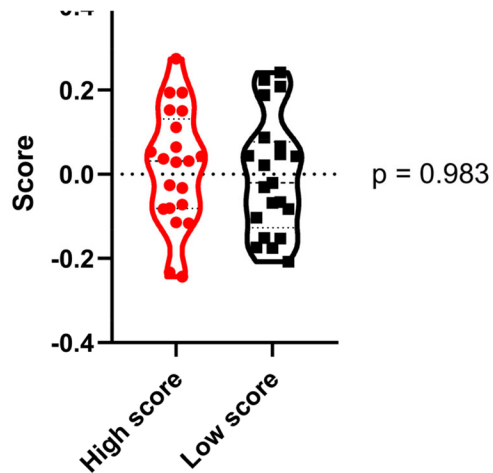

Supplement: Supplementary file 7 — Additional file 7: Fig. S7. Immunophenscore of high- and low-score group. No significant difference found between groups. A) Immunophenscore (EC) of high- and low-score group. B) Immunophenscore (CP) of high- and low-score group. C) Immunophenscore (SC) of high- and low-score group. [file 13058_2022_1516_MOESM7_ESM.pdf]

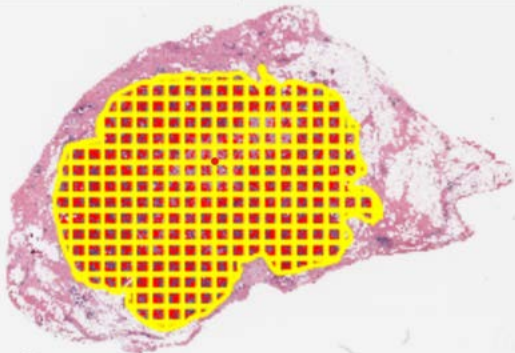

Supplement: Supplementary file 10 — Additional file 10: Fig. S10. The illustration of ROI segmentation on H.E slice. [file 13058_2022_1516_MOESM10_ESM.pdf]
