# Supplementary material for: Radiomics predicts the prognosis of patients with locally advanced breast cancer by reflecting the heterogeneity of tumor cells and the tumor microenvironment
Source: Breast Cancer Res. 2022 Mar 15;24:20. doi: 10.1186/s13058-022-01516-0 (PMC8922933; doi:10.1186/s13058-022-01516-0)
Supplement: Supplementary file 4 — Additional file 4: Fig. S4. Calibration plot of validation cohort. [file 13058_2022_1516_MOESM4_ESM.pdf]

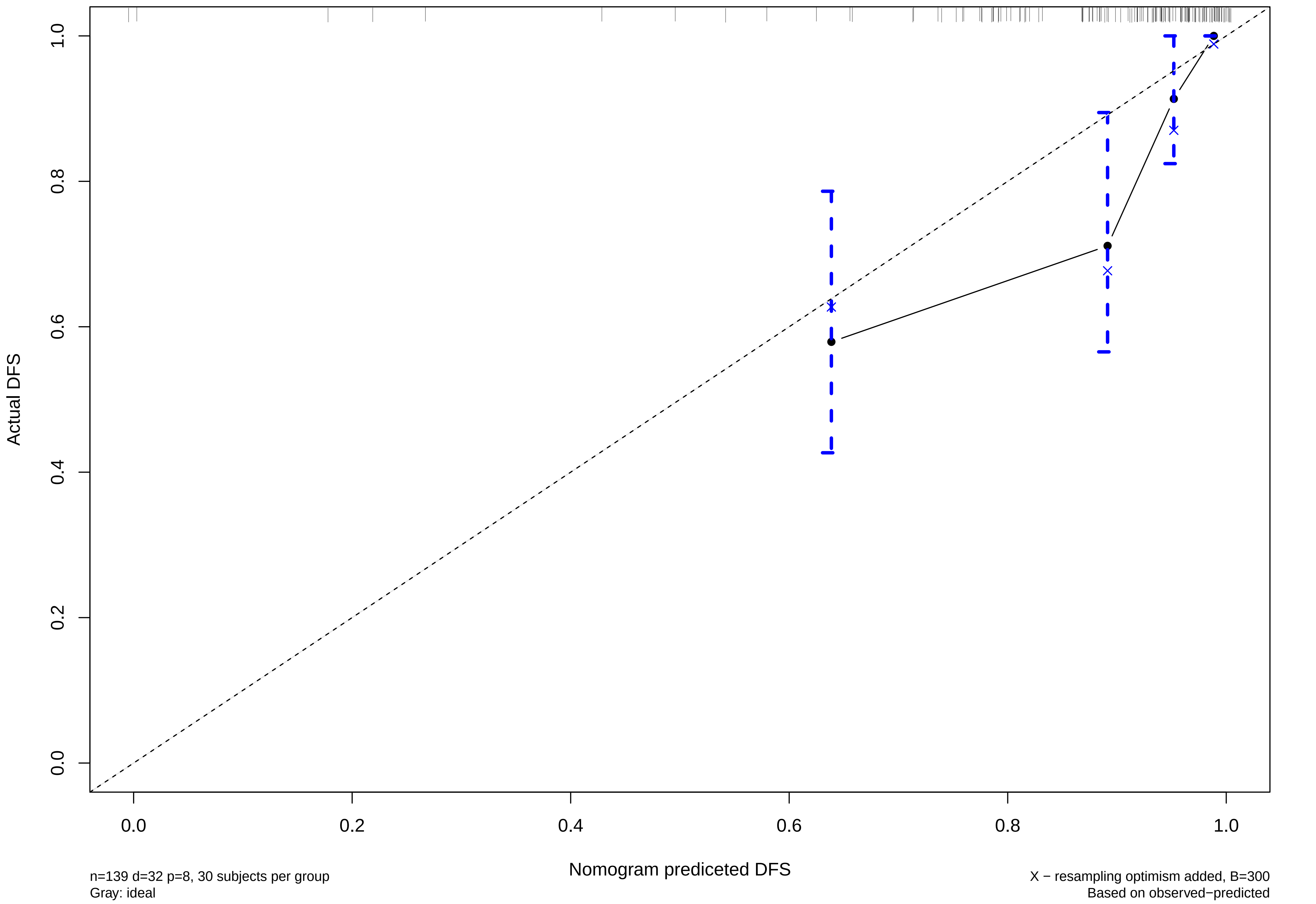

1.0
0.8
0.6
0.4
0.2
0.0
Actual DFS
0.0
0.2
0.4
0.6
0.8
1.0
Nomogram prediceted DFS
n=139 d=32 p=8, 30 subjects per group
Gray: ideal
X − resampling optimism added, B=300
Based on observed−predicted
