# Supplementary material for: Radiomics predicts the prognosis of patients with locally advanced breast cancer by reflecting the heterogeneity of tumor cells and the tumor microenvironment
Source: Breast Cancer Res. 2022 Mar 15;24:20. doi: 10.1186/s13058-022-01516-0 (PMC8922933; doi:10.1186/s13058-022-01516-0)
Supplement: Supplementary file 5 — Additional file 5: Fig. S5. The workflow of the association between tumor heterogeneity and radiomics. Heterogeneity which stems from tumor cell and microenvironment is evaluated. [file 13058_2022_1516_MOESM5_ESM.pdf]

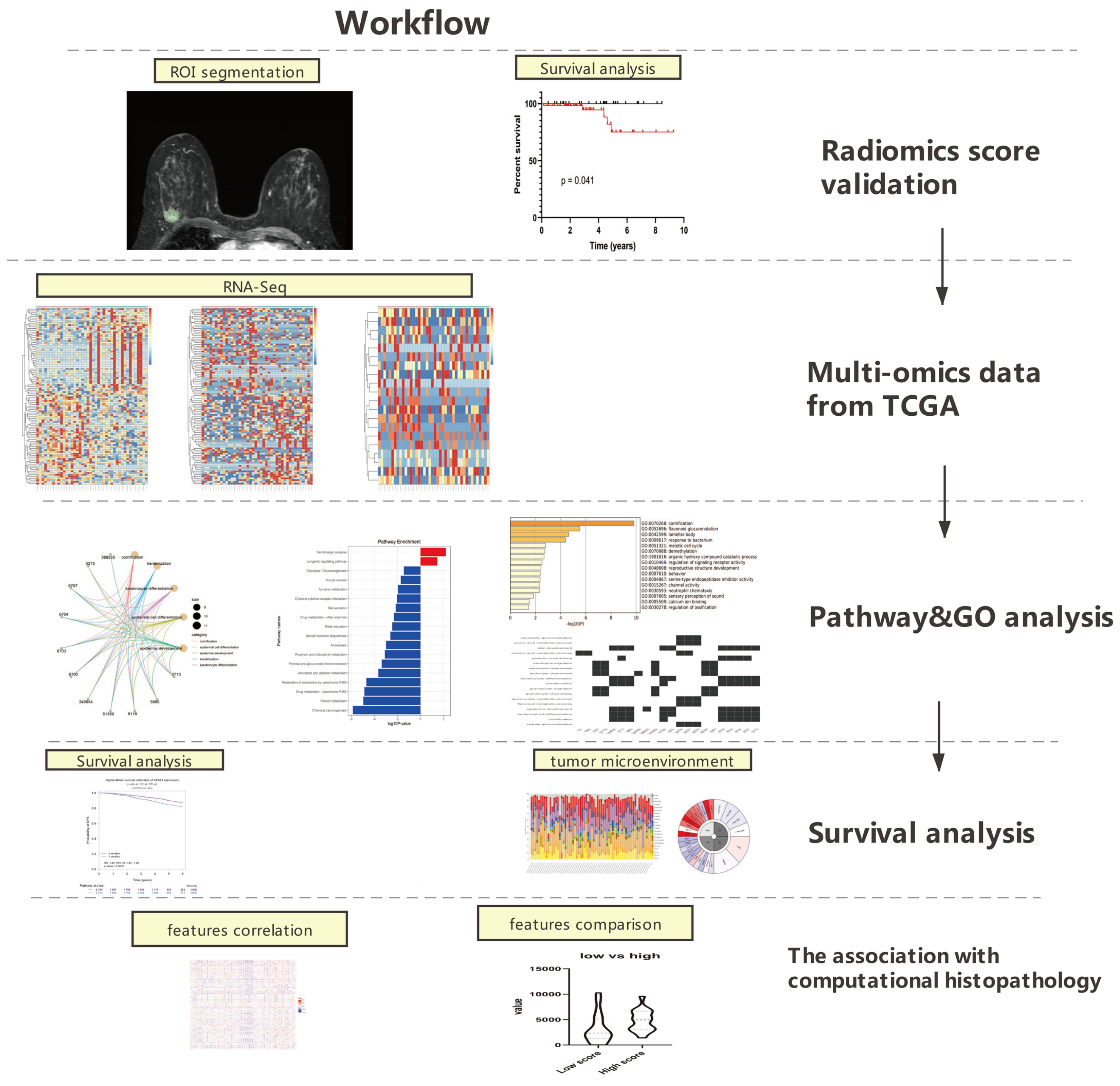

Workflow
ROI segmentation
Survival analysis
100
50
0
Percent survival
p = 0.041
0 2 4 6 8 10
Time (years)
Radiomics score validation
RNA-Seq
Multi-omics data from TCGA
Pathway&GO analysis
Survival analysis
tumor microenvironment
Survival analysis
features correlation
features comparison
low vs high
15000
10000
5000
0
value
Low score
High score
The association with computational histopathology
