# Supplementary material for: Radiomics predicts the prognosis of patients with locally advanced breast cancer by reflecting the heterogeneity of tumor cells and the tumor microenvironment
Source: Breast Cancer Res. 2022 Mar 15;24:20. doi: 10.1186/s13058-022-01516-0 (PMC8922933; doi:10.1186/s13058-022-01516-0)
Supplement: Supplementary file 8 — Additional file 8: Fig. S8. The radiomics features extracted in this study using “slicer radiomics”. [file 13058_2022_1516_MOESM8_ESM.pdf]

Feature class	Feature name									
Shape	Elongation	Flatness	LeastAxisLength	MajorAxisLength	Maximum2DDiameterColumn	Maximum2DDiameterRow	Maximum2DDiameterSlice	Maximum3DDiameter	MeshVolume	MinorAxisLength
	Sphericity	Surface Area	Surface Volume Ratio	VoxelVolume						
First-order	10Percentile	90Percentile	Energy	InterquartileRange	Kurtosis	Maximum	MeanAbsoluteDeviation	Mean	Median	Minimum
	Range	Robust MeanAbsoluteDeviation	RootMeanSquared	Skewness	TotalEnergy	Variance				
GLDM	DependenceEntropy	DependenceNonUniformity	DependenceNonUniformityNormalized	DependenceVariance	GrayLevelNonUniformity	LargeDependenceEmphasis	LargeDependenceHighGrayLevelEmphasis	LargeDependenceLowGrayLevelEmphasis	SmallDependenceEmphasis	SmallDependenceHighGrayLevelEmphasis
	SmallDependenceLowGrayLevelEmphasi									
GLRLM	GrayLevelNonUniformity	LongRunEmphasis	LongRunHighGrayLevelEmphasis	LongRunLowGrayLevelEmphasis	RunEntropy	RunLengthNonUniformity	RunLengthNonUniformityNormalized	RunPercentage	RunVariance	ShortRunEmphasis
	ShortRunEmphasis	ShortRunLowGrayLevelEmphasis								
GLSZM	GrayLevelNonUniformity	LargeAreaEmphasis	LargeAreaHighGrayLevelEmphasis	LargeAreaLowGrayLevelEmphasis	SizeZoneNonUniformity	SizeZoneNonUniformityNormalized	SmallAreaEmphasis	SmallAreaHighGrayLevelEmphasis	SmallAreaLowGrayLevelEmphasis	ZoneEntropy
	ZonePercentage	ZoneVariance								
Wavelet-based	Wavelet transformation based on above features.									
