# Supplementary material for: Radiomics predicts the prognosis of patients with locally advanced breast cancer by reflecting the heterogeneity of tumor cells and the tumor microenvironment
Source: Breast Cancer Res. 2022 Mar 15;24:20. doi: 10.1186/s13058-022-01516-0 (PMC8922933; doi:10.1186/s13058-022-01516-0)
Supplement: Supplementary file 9 — Additional file 9: Fig. S9. The computational histopathological features in this study. [file 13058_2022_1516_MOESM9_ESM.pdf]

Feature class	Feature name							
Shape	Area	Length	Circularity	Solidity	Maximum diameter	Minimum diameter	Nucleus/Cell area ratio	
Intensity	Mean	Min	Max	Standard deviation	Mean	Haralick features		
Smooth								
Tumor cell	Centroid	Nucleus Area	Nucleus Perimeter	Nucleus Circularity	Nucleus Max caliper	Nucleus Min caliper	Nucleus Eccentricity	Nucleus Hematoxylin OD
	Nucleus Eosin OD	Cell Area	Cell Perimeter	Cell Circularity	Cell Max caliper	Cell Min caliper	Cell Eccentricity	Cell Hematoxylin OD
	Cell Hematoxylin OD	Cytoplasm Hematoxylin OD	Cytoplasm Eosin OD	Nucleus Cell area ratio				
